# Fluoxetine normalizes disrupted light-induced entrainment, fragmented ultradian rhythms and altered hippocampal clock gene expression in an animal model of high trait anxiety- and depression-related behavior

**DOI:** 10.3109/07853890.2015.1122216

**Published:** 2015-12-18

**Authors:** Jörg Schaufler, Marianne Ronovsky, Giorgia Savalli, Maureen Cabatic, Simone B. Sartori, Nicolas Singewald, Daniela D. Pollak

**Affiliations:** ^a^ Department of Neurophysiology and Neuropharmacology, Center for Pharmacology and Physiology, Medical University of Vienna, Vienna, Austria; ^b^ Department of Pharmacology and Toxicology, Institute of Pharmacy and CMBI, Leopold-Franzens-University of Innsbruck, Innsbruck, Austria

**Keywords:** Anxiety disorders, circadian rhythm, clock genes, depression, fluoxetine, hippocampus, mouse model

## Abstract

**Introduction** Disturbances of circadian rhythms are a key symptom of mood and anxiety disorders. Selective serotonin reuptake inhibitors (SSRIs) - commonly used antidepressant drugs – also modulate aspects of circadian rhythmicity. However, their potential to restore circadian disturbances in depression remains to be investigated.

**Materials and methods** The effects of the SSRI fluoxetine on genetically based, depression-related circadian disruptions at the behavioral and molecular level were examined using mice selectively bred for high anxiety-related and co-segregating depression-like behavior (HAB) and normal anxiety/depression behavior mice (NAB).

**Results** The length of the circadian period was increased in fluoxetine-treated HAB as compared to NAB mice while the number of activity bouts and light-induced entrainment were comparable. No difference in hippocampal *Cry2* expression, previously reported to be dysbalanced in untreated HAB mice, was observed, while *Per2* and *Per3* mRNA levels were higher in HAB mice under fluoxetine treatment.

**Discussion** The present findings provide evidence that fluoxetine treatment normalizes disrupted circadian locomotor activity and clock gene expression in a genetic mouse model of high trait anxiety and depression. An interaction between the molecular mechanisms mediating the antidepressant response to fluoxetine and the endogenous regulation of circadian rhythms in genetically based mood and anxiety disorders is proposed.

## Introduction

Psychiatric illnesses including mood and anxiety disorders are often associated with dysregulation of circadian rhythms, such as sleep disturbances (DSM-V 2013; ICD-10 1992, Version 2015) (1,2). Reversely, chronic disruptions of sleep patterns, like those resulting from shift-work or repetitive jet-lags constitute a risk factor for the development of these illnesses (3,4). An involvement of the endogenous circadian machinery in mood and anxiety disorders is further supported by several lines of evidence derived from genetic association studies reporting specific polymorphisms of clock genes in the respective patient populations (5–12). Whether and how dysregulation of the circadian system is causally and mechanistically involved in the pathogenesis of these disorder remains incompletely understood, substantially due to the lack of appropriate animal models.

The most commonly prescribed drugs in the pharmacotherapy of mood and anxiety disorders exert their effects through modulation of monoaminergic neurotransmission. Specifically, selective serotonin reuptake inhibitors (SSRIs), such as fluoxetine, target the serotonergic system by blocking the reuptake of serotonin from the synaptic cleft through their inhibitory effect on the serotonin transporter, hence augmenting the amount of free serotonin available. Notably, the serotonergic system, central to the neural circuitry of mood and anxiety disorders (13,14) and the circadian system, are extensively and reciprocally linked at anatomical and molecular levels. Furthermore, there is genetic, physiological, and clinical evidence that these systems converge in the joint regulation of emotional and circadian behaviors and that perturbations in both systems are associated with mood disorders (15). Indeed, SSRIs have been found to modulate circadian rhythmicity after acute (16,17), but not after chronic exposure (18). These effects have been attributed mainly to functional and molecular alterations in the suprachiasmatic nucleus (SCN) (16,17,19,20), the master pacemaker in the brain, orchestrating the rhythmicity of the endogenous circadian clock. Apart of the SCN, many other brain areas exhibit clock gene expression, including the hippocampus – a critically implicated in the neural circuitry of depression (21) where effects of fluoxetine administration on clock gene expression have been described (22).

Yet, the potential of SSRIs to restore circadian disturbances in depression remains to be investigated. The determination of the effects of a most widely used pharmacological antidepressant on circadian rhythms at the behavioral and molecular level may be an important step forward into the elucidation of the intricate involvement of the circadian system in the pathophysiology of depression and its therapeutic management. To address this question, we here employed a validated animal model, mice selectively bred for trait anxiety and co-morbid depression (HAB) and their normal trait anxiety and depression controls (NAB). We aimed to explore whether the disrupted behavioral circadian rhythms and alterations in hippocampal clock gene expression previously observed in HAB mice (23) can be ameliorated by chronic fluoxetine treatment. Female mice were selected for the present study, since the behavioral and cellular alterations related to depression have been shown to be reversible by fluoxetine treatment in female but not male HAB mice (24,25). The chronic administration regime is in accordance with the protocol previously employed (24,25) and constitutes a paradigm paralleling the human situation, where long-term SSRI treatment is required for clinical efficacy,

## Materials and methods

### Animals

Experiments were carried out in adult, female HAB and NAB mice obtained from the Department of Pharmacology and Toxicology, University of Innsbruck, Austria. The anxiety-related phenotype was verified by an Elevated Plus-Maze test at seven weeks of age as previously described (26) (Supplementary Figure S1). At the initiation of circadian locomotor analysis mice were 15–20 weeks of age with an average body weight (g) of 29.22 ± 0.44. All experiments were designed to minimize animal suffering and the number of animals used. Animal procedures were approved by the Austrian ethical committee (BMWF-66.009/0302-II/36/2013) on animal care and use and conducted in accordance with international laws and policies.

### Housing

Mice were housed individually in Nalgene cages equipped with running wheels (15 cm in diameter; Actrimetrics, Evanston, IL) in a sound-attenuated room with constant temperature of 23 ± 2 °C. Before experimental assessment of the circadian activity all animals were kept on a 12 h: 12 h light: dark cycle (LD). During the light phase, mice were exposed to a light intensity of ∼200  lux. During conditions of constant darkness constituting a 0h:24h light: dark cycle (DD), cage cleaning and animal care taking was carried out under dim red light (15 W). Mice were supplied *ad libitum* with food and fluoxetine-containing tap water according to the experimental design (Figure 1).

**Figure:**
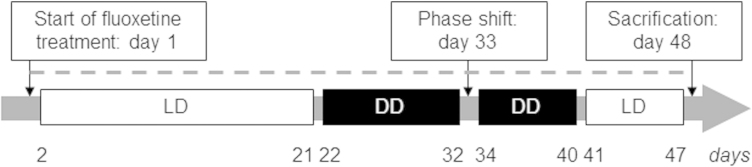
**Figure 1.** Experimental procedure for the assessment of the effects of chronic fluoxetine treatment on behavioral and molecular parameters of the circadian clock in HAB and NAB mice. Depicted is the time course (in days) of drug administration (dashed line) and respective light regimes light/dark (LD): 12h light and 12h dark phase, white boxes; dark/dark (DD): 24 h constant darkness, black boxes) for the experimental evaluation of the effects of chronic fluoxetine treatment on circadian wheel-running activity and hippocampal clock gene expression in female mice selectively bred for high (HAB) and normal (NAB) anxiety-related and depression-like behavior.

### Drug treatment

Fluoxetine hydrochloride (Sigma Aldrich, Vienna, Austria) was administered via the drinking water at a dose (18 mg kg^−1^ day^−1^) previously described to reverse depression-like behavior in female HAB mice (24). The concentration of the drug in water was adapted based on the individual daily liquid consumption (determined twice a week) and body weight of each animal (evaluated weekly).

### Assessment of circadian wheel-running activity

#### Acquisition

Wheel revolutions were recorded using the ClockLab computer software, with sampling epochs of 1 min (Actimetrics, Evanston, IL). One day after the initiation of fluoxetine treatment, the light-entrained circadian activity was assessed for 20 days during LD followed by the evaluation of the free-running circadian activity during DD. On day 33 DD was briefly interrupted by a light pulse (30 min, 300 lux) at circadian time (CT) 16 (four hours after activity onset) for the induction of a phase shift in order to evaluate the response of the endogenous circadian pacemakers to external zeitgebers. After eight more days of DD all mice were exposed to LD for nine days before scarification on day 48 (Figure 1).

#### Analysis

Wheel-running activity was analyzed using the ClockLab software package (Actimetrics, Evanston, IL) as previously described (23). The default software settings were used to determine the activity onsets which were manually edited when appropriate. Measures of the circadian period (*tau*) and the total activity were derived from regression lines fit to the activity onsets. Activity bouts were defined as periods during which activity never reached less than one count per min (bout threshold) for longer than 18 min (maximum gap length) at a time. All parameters were determined for each animal under LD and DD conditions. Phase shift responses were evaluated by comparing the predicted activity onset for the day after the light pulse treatment from extrapolated lines of the activity onsets of the days preceding the light pulse and seven days after the pulse.

### Gene expression analysis

#### Brain dissection

All brain dissections were carried out during the light-phase of the circadian cycle (between 9 a.m. and 11 a.m.). Mice were deeply anesthetized by the i.p. administration of a ketamine/xylazine (100 mg kg^−1^/10 mg kg^−1^) combination and exsanguinated by cardiac puncture at the time of sacrifice. Brains were rapidly dissected over ice and total hippocampi were bilaterally collected and stored in RNA later ® (Ambion, Austin, TX) at −20 °C until used for RNA isolation or kept at −80 °C for protein expression studies.

#### RNA isolation, cDNA synthesis, quantitative real-time polymerase chain reaction (qRT-PCR)

RNA was isolated from hippocampal tissues using the miRNeasy kit (Qiagen®, Hilden, Germany) in adherence to the manufacturer’s instructions. Briefly, 900 ng of total RNA was used for cDNA synthesis using the mMLV reverse transcriptase first-strand cDNA synthesis kit, G1 (Biozym®, Hessisch Oldendorf, Germany) following the manufacturer’s instructions provided. The resulting cDNA reaction mix (1:10 dilution) was used for PCR amplification using the Fast SYBR Green Mastermix (Applied Biosystems, Foster City, CA) on a StepOnePlus real-time PCR system (serial no. 271000455; Applied Biosystems, Foster City, CA). All reactions were carried out in duplicates. Primer sequences for all clock genes analyzed (Circadian locomotor output cycles kaput (Clock), Brain and muscle aryl hydrocarbon receptor nuclear translocator-like 1 (Bmal1), Period 1-3 (Per1-3), Cryptochrome 1/2 (Cry1/2), RAR (Retinoic Acid Receptor)-related orphan receptor α-γ (Rorα-γ), Reverse erythroblastosis virus α/β (Rev-erbα/β), and Neuronal PAS domain-containing protein 2 (Npas2) are listed in (23) Supplementary Table 1).

**Table 1.** Unaltered mRNA expression of clock genes and clock-controlled genes in the hippocampus of NAB versus HAB mice under chronic fluoxetine treatment.

mRNA	NAB	HAB	p value
*Clock*	1.000 ± 0.024	0.979 ± 0.061	0.7615
*Bmal1*	1.000 ± 0.037	0.919 ± 0.080	0.3758
*Per1*	1.000 ± 0.119	1.070 ± 0.064	0.6155
*Cry1*	1.000 ± 0.054	1.156 ± 0.104	0.2054
*Cry2*	1.000 ± 0.015	1.039 ± 0.062	0.5545
*Rorα*	1.000 ± 0.110	1.285 ± 0.218	0.2847
*Rorβ*	1.000 ± 0.026	1.028 ± 0.114	0.8153
*Rorγ*	1.000 ± 0.815	1.048 ± 0.148	0.8378
*Rev-erbα*	1.000 ± 0.056	0.991 ± 0.110	0.9429
*Rev-erbβ*	1.000 ± 0.094	1.021 ± 0.086	0.8739
*Npas2*	1.000 ± 0.067	0.991 ± 0.146	0.9572

Mean HAB fold-change values (n = 7–8 per group) are normalized to NAB means for each gene analyzed and are displayed as mean ± SEM. p values derived from statistical comparisons using two-tailed Student-*t-*tests are provided.

The C(t) values of β-actin were used for calculation of ΔC(t), representing the relative quantification of mRNA amounts in each sample. This further allowed the calculation of ΔΔC(t), subtracting mean ΔC(t) value of the NAB from the mean ΔC(t) value for the HAB. ΔΔC(t) was then used to express the fold change of mRNA levels observed between HAB and NAB mice, using the formula 2^-ΔΔC(t)^.

#### Protein isolation and western blotting

Hippocampal tissue was powderized in liquid nitrogen and homogenized in a protein lysis buffer containing 10 mM Tris-HCl, pH 7.5, 150 mM NaCl, 1% SDS, 0.5% Triton X100, 1 mM EDTA, 10 mM NaF, 5 mM Na4O2P7, 10 mM Na3VO4 and protease inhibitor cocktail (1×, Roche Diagnostics, Mannheim, Germany). After sonication for approximately five cycles × 5s × 5, the suspension was left at 4 °C on a rotator for 30 minutes and centrifuged at 1 4000 × g for 30 min at 4 °C. The supernatant was immediately transferred and was quantified using Pierce BCA assay Kit (Thermo Scientific, Vienna, Austria). The standard curve was generated using Bovine Serum Albumin ampules with a concentration of 2 mg/ml. The samples were analyzed in triplicate (microplate procedure: 25 μl sample + 200 μl BCA working reagent and incubated at 37 °C for 30 minutes) and the concentration was determined by absorbance reading at 595 nm using a Synergy H4 Hybrid Reader spectrophotometer (Szabo-Scandic HandelsgmbH & Co KG, Vienna, Austria).

Samples (25μg protein) were analyzed and loaded on a 10% sodium dodecyl sulfate (SDS) mini-gel (0.75 mM × 6.8 cm × 8.6 cm) and 5% stacking gel and then subjected to electrophoresis at 80 V for 1 hour and 45 min. Electrophoresis was performed with a Mini-Protean System (Bio-Rad Laboratories Inc., Vienna, Austria). Proteins from the gel were transferred onto PVDF membranes (Millipore, MA) and run at 250 mA for 1h 30 minutes. Membranes were blocked by incubating with 5% nonfat dry milk in 100 mM Tris, pH 7.5, 150 mM NaCl, and 0.1% Tween 20 (TTBS) for 1h. Membranes were then incubated with a diluted primary antibody (Per2 and Per3 (Alpha Diagnostic International Inc, TX) [1:1000], Cry2 (Abcam, Plc, Cambridge, UK) [1:500]) overnight at 4 °C, rinsed three times with TTBS, and incubated for 1h at room temperature with a horseradish peroxidase-conjugated secondary antibody (Goat Anti-rabbit HRP-linked IgG, Cell Signaling Technology, Inc., Danvers, MA [1:3000]). Immunoreactivity was visualized by enhanced chemiluminescence Pierce ECL substrate (Thermo Scientific, Vienna, Austria). Detectable molecular masses were determined by running standard protein markers (Thermo Scientific, Vienna, Austria) ranging from 10 to 250 kDa. Quantification was performed by chemiluminescent imaging with a FluorChem HD2 (Alpha Innotech, San Leandro, CA) using the respective software. Values obtained from densitometry of the target proteins were normalized to the housekeeping protein β-tubulin for the same samples.

### Statistical analysis

BioStat software (AnalystSoft Inc., Alexandria, VA,) was used for statistical analysis. Comparisons were determined using unpaired two-tailed Student’s *t*-test. The level of significance was set at p < 0.05 in all instances.

## Results

### Chronic fluoxetine treatment exerts selective effects on behavioral circadian locomotor rhythms in HAB mice

In order to investigate whether genetically based trait anxiety and co-morbid depression determine potential effects of the SSRI fluoxetine on circadian behavior, wheel-running activity rhythms were compared between HAB and NAB mice under chronic fluoxetine treatment.

**Figure 2.**
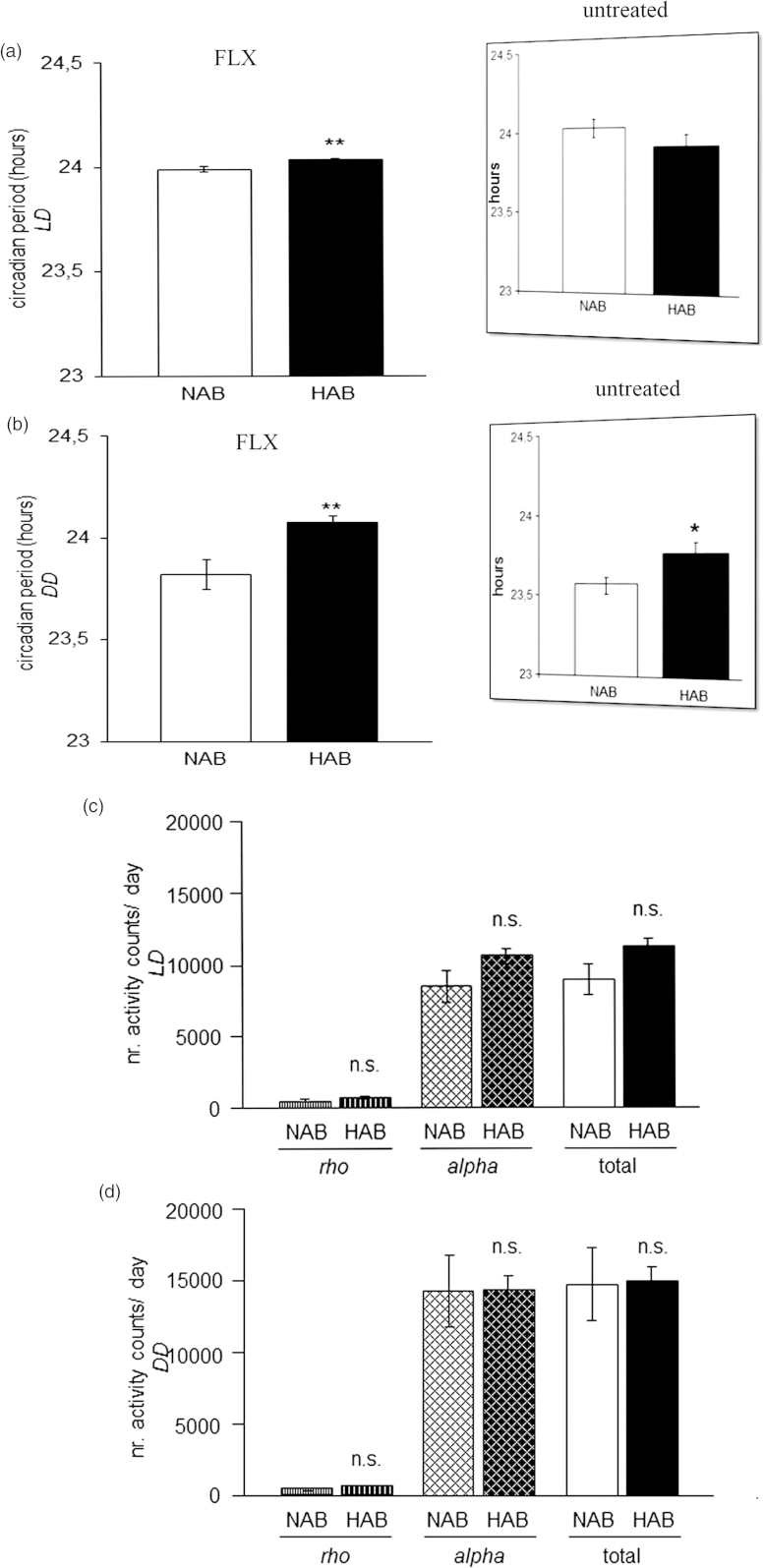
Circadian period *(tau)* and wheel-running activity rhythms in fluoxetine-treated HAB and NAB mice. During chronic fluoxetine treatment HAB mice showed a longer circadian period (*tau*) than NAB mice both under (a) light-entrained (LD) and under (b) free-running (DD) conditions as compared with no treatment (previously reported in (23) and reprinted here in inserts (with permission from Annals of Medicine). No differences in the *total* amount of wheel-running activity per day between HAB and NAB mice was detected, nor during either their active (*alpha*) or inactive (*rho*) under (c) light-entrained and (d) free-running conditions (n= 8–16 per group). **p < 0.01, n.s. (not significant), p > 0.05. All data are displayed as mean ± SEM.

**Figure 3.**
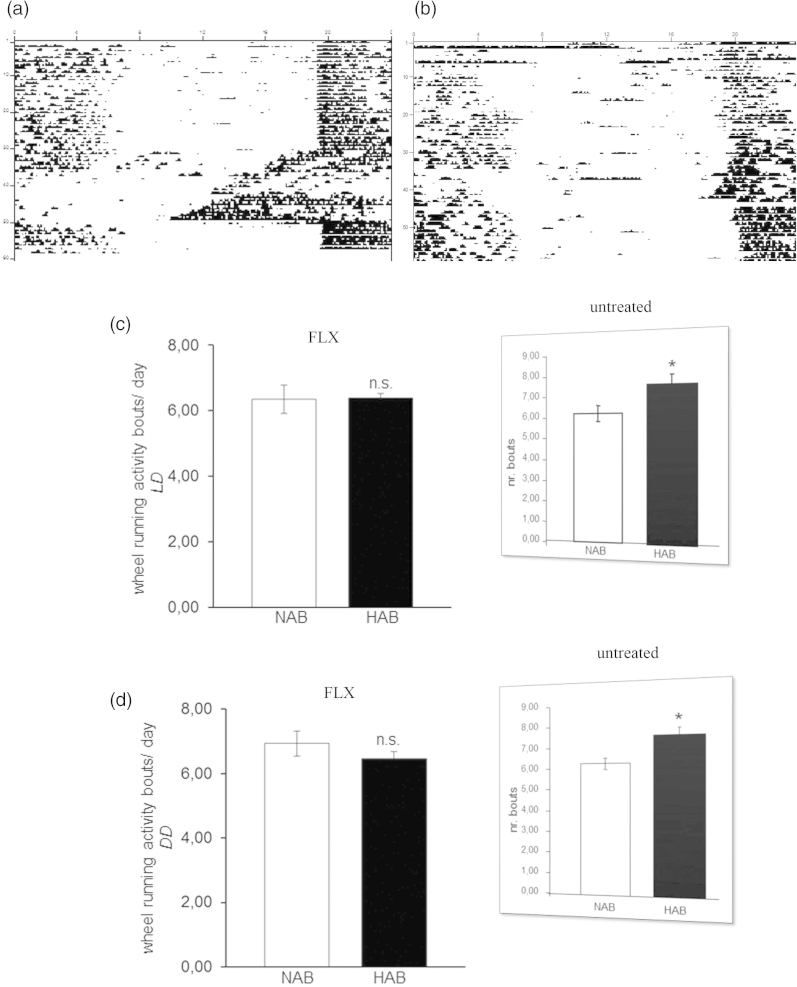
Circadian wheel-running activity bouts in HAB and NAB mice under chronic fluoxetine treatment. Sample actograms illustrating circadian wheel-running activity in chronically-fluoxetine treated (a) NAB and (b) HAB mice. NAB and HAB mice show comparable numbers of daily wheel-running activity bouts under (c) light-entrained (LD) and (d) free-running conditions (DD) (n= 8–16 per group). Inserts (reprinted with permission from Annals of Medicine) represent previously obtained results in untreated HAB and NAB mice (23). n.s. (not significant) p > 0.05. All data are displayed as mean ± SEM.

The circadian period (*tau*) was longer in HAB than NAB mice under both LD (p = 0.001; Figure 2a) and DD conditions (p = 0.001; Figure 2b), indicating that fluoxetine acted to enhance the previously described elongations in *tau* in untreated HAB mice, irrespective of the light condition (results from (23) are depicted in inserts in Figures 2a and b). The daily amount of wheel-running activity was comparable between HAB and NAB mice during inactive (*rho*) and active phases (*alpha*) of the circadian cycle under both LD (p > 0.05, Figure 2c) and DD (p > 0.05, Figure 2d) conditions suggesting that the significant differences in *tau* do not result from alterations in overall locomotor activity. In order to examine a potential effect of fluoxetine treatment on the ultradian structure of circadian profiles in HAB and NAB mice, the number of activity bouts per day was evaluated. No evidence for differential fragmentation of circadian rhythms in HAB and NAB mice upon fluoxetine treatment (see representative actograms Figure 3a and b) were obtained, as the number of daily activity bouts was comparable in HAB and NAB mice both under LD (p > 0.05, Figure 3c) and DD conditions (p > 0.05, Figure 3d). A significant enhancement in the number of daily activity bouts had been observed in untreated HAB mice in an earlier report [results from (23)] are depicted as inserts in Figures 3a and b). In order to shed light on the adaptability of the endogenous circadian regulatory system to external *Zeitgeber* under fluoxetine treatment, light-induced entrainment was assessed in HAB and NAB mice by calculation of the phase-shift response upon exposure to a brief light pulse at CT14 under DD conditions. Both HAB and NAB mice responded with a phase delay which was in magnitude a match for what was expectable according to previous reports from literature using the same protocol (p > 0.05, Figure 4a) hence blunting the previously described differences in untreated animals [results from (23) are depicted in inserts in Figure 4a].

**Figure:**
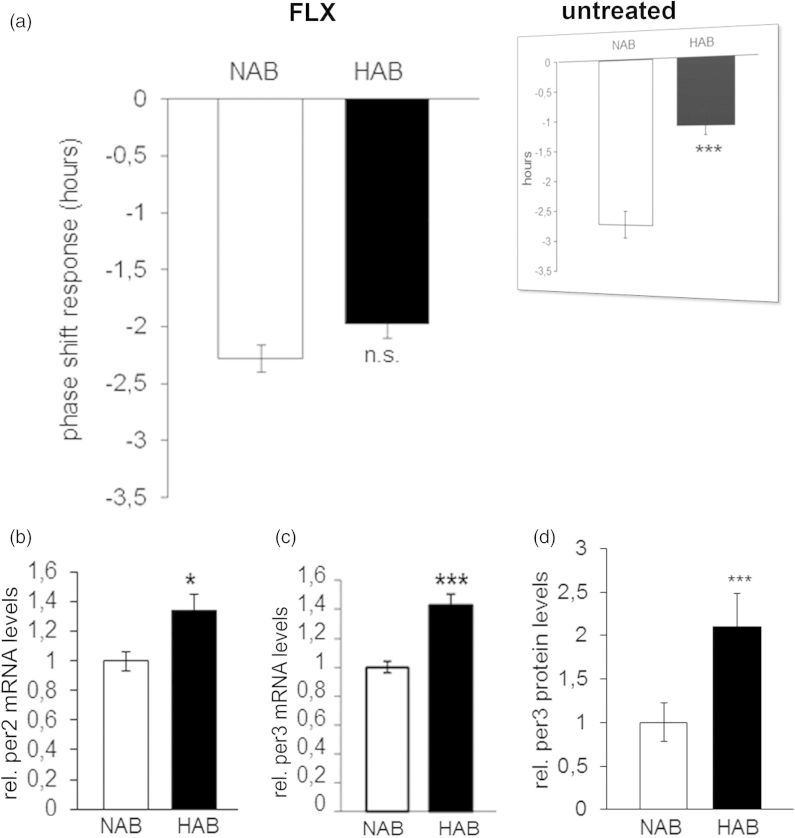
**Figure 4.**Phase shift response and relative hippocampal clock gene expression in HAB and NAB mice chronically treated with fluoxetine. (a) The phase shift response upon exposure to a brief light pulse at CT14 is comparable in HAB and NAB mice (n= 8–16 per group). Inserts (reprinted with permission from Annals of Medicine) represent previously obtained results in untreated HAB and NAB mice (23). (b) Per2 and (c) Per3 mRNA expression and (d) Per3 protein expression is significantly higher in hippocampal tissue of fluoxetine-treated HAB as compared to NAB mice (n = 7–8 per group). *p < 0.05, ***p < 0.001. All data are data displayed as mean ± SEM.

### Differential hippocampal clock gene expression in HAB and NAB mice after chronic fluoxetine treatment

Aiming to identify potential molecular correlates of the circadian behavioral phenotypes described above, mRNA expression of 13 core clock genes (*clock*, *bmal1, per1-3*, *cry1/2*, *rorα-γ*, *rev-erbα/β* and *npas2*) was analyzed by qRT-PCR in hippocampal tissue of fluoxetine-treated HAB and NAB mice. SYBR Green was used as fluorescent DNA binding dye to determine the rate amplification of the target gene relative to a house keeping gene (beta-actin) for each sample. Expressional levels of hippocampal *per2* and *per3* were significantly higher in HAB than in NAB mice after chronic fluoxetine treatment (p < 0.05 and p < 0.001; Figure 4b and c). No differences in the mRNA levels of *cry2*, previously found to be higher expressed in drug-naïve HAB as compared to NAB mice (23) or any of the other clock genes examined (p > 0.05; Table 1) was observed. Augmented expression of per3 was also observed at the protein level in hippocampal tissue of fluoxetine-treated HAB mice (p < 0.05; Figure 4d) while levels of per2 and cry2 did not alter significantly between HAB and NAB (Supplementary Figure S2). Similarly, only *per3*, but not *per2* or *cry2* mRNA was differentially expressed in prefrontal cortical tissue of fluoxetine-treated HAB as compared to NAB mice (Supplementary Figure S2).

## Discussion

We here provide first evidence that fluoxetine treatment normalizes disrupted circadian rhythms and altered hippocampal clock gene expression in a genetic animal model of high trait anxiety and depression. Although fluoxetine was supplied via the drinking water, no fluctuation of liquid consumption over the treatment period was found (data not shown). While this observation suggests that – in contrast to previous reports (27,28) – fluoxetine treatment did not induce an initial taste aversion in either HAB or NAB mice, the possibility of a reduction in liquid intake resulting from fluoxetine treatment cannot be definitively excluded.

In comparison to an earlier report examining drug-naive animals (23), chronic fluoxetine treatment differentially modulated selected parameters of the circadian activity profile in HAB and NAB mice. Thus, it is feasible to propose that some of the previously reported alterations in circadian behavior in untreated HAB as compared to NAB mice, including the fragmentation of the ultradian rhythm and the aberrant light-induced phase shift response entrainment were rescued by chronic SSRI treatment. In contrast, the alteration in the length of the circadian period (*tau*) under free-running conditions previously described in drug-naïve HAB mice (23) remained unaffected by fluoxetine treatment suggesting that fluoxetine, which efficiently reduces anxiety and depression-like behavior in female HAB mice, is not suitable to correct for the associated aberrations in the duration of the circadian period. Fluoxetine also had no impact on light-entrained *tau*, since neither in NAB nor in HAB the length of the daily period observed in the present study was different from previous observations (23).

By contrast, the fragmentation of the ultradian rhythm in untreated HAB versus NAB mice which was previously indicated by an enhanced number of activity bouts (23) and alterations in sleep architecture (29) was not longer observable under the influence of chronic fluoxetine exposure. The potential translational value of this finding could be tested by examining whether fluoxetine treatment might exert a beneficial effect on aberrant ultradian rhythms, including disrupted sleep architecture, in a specific subgroup of mood disorder patients presenting with a particular genetic profile, potentially characterized by variants in the sequence and/or expression of one or more clock genes (5–12).

When exposed to a brief light pulse in the early subjective night (CT 16), HAB and NAB mice chronically treated with fluoxetine exhibit a comparable phase shift response, implying that fluoxetine treatment efficiently restored the deficient light-induced resetting of the behavioral circadian rhythm displayed by untreated HAB mice (23). Interestingly, acute administration of fluoxetine has been reported to modulate light-induced phase shift responses *in vitro* (17) and *in vivo* in hamsters (30), rats (19) and mice (31) whereas chronic administration of fluoxetine does not seem to regulate photic entrainment in both young and aged hamster (18). However, to the best of our knowledge, this is the first study investigating the effects of long-term fluoxetine treatment on light-dependent resetting of the endogenous circadian rhythm in an animal model of anxiety and comorbid depression. Considering that the present study used female mice and estrus stages were not determined in the experimental animals, the impact of the menstrual cycle and its interaction with drug treatment in HAB and NAB mice remains to be investigated.

In search for potential molecular correlates of the observed differences in the adaptation of the circadian locomotor phenotype paralleling long-term fluoxetine exposure, we analyzed the expression of 13 core clock genes in the hippocampus of HAB and NAB mice. The hippocampus is a brain region highly implicated in the neural circuitry of mood and anxiety disorders (32) and is dysfunctional in HAB animals in response to stress (24,25). Fluoxetine increased the hippocampal expression of *per2* and *per3* in HAB versus NAB mice while no differences in *per2* and *per3* mRNA levels in untreated HAB and NAB animals had been previously described. *Per2* and *per3* are essential elements of the auto-regulatory feed-back loops underlying the generation and control of circadian rhythms. By forming heterodimers with the *Cry* proteins they act to inhibit their own transcription through binding to the *Clock:Bmal1* complex (33). Interestingly, fluoxetine treatment apparently acted to normalize the reduction of levels of *cry2* mRNA in the hippocampus, characteristic of drug-naïve HAB mice (23).

Studies in humans have yielded evidence for an association of genetic variants of *per2*, *per3* and *cry2* with mood disorders such as SAD (seasonal affective disorder) (8,10), bipolar disorder and dysthymia (6), respectively selective characteristics of these conditions (7,34,35–37). In an experimental setting a reduction in the expression of *per2* has been observed in the mouse hippocampus in a chronobiological animal model of depression (38) whereas antidepressant pharmacotherapy increases levels of *per2* in a time of day-dependent manner (39). Interestingly, *per2* also seems to be involved in regulating adult hippocampal neurogenesis (40,41), a particularly relevant result in light of the well validated link between adult hippocampal neurogenesis and the therapeutic effects of SSRIs (42), which has also previously been examined in HAB mice (43). Indeed, a distinctive profile of hippocampal clock gene expression, including levels of *per2, per3* and *cry2* induced by fluoxetine and cocaine treatment respectively, has been previously described (22). As for *per3*, a loss of diurnal rhythmicity has been recently reported in a chronic stress based mouse model of depression, focusing on mRNA levels in the amygdala (44). Hippocampal expression of *per3* has been tightly correlated to anxiety-related behaviors and modulation by stress-exposure proposes an important function of forebrain *per3* in the regulation of emotional states (45).

In the earlier study comparing circadian rhythms in untreated HAB versus NAB lines, a selective reduction in the levels *cry2* has been observed in hippocampal tissue of HAB mice (23). Indeed, *cry2*-knock-out mice have been found to present with augmented anhedonic behavior (46). This observation lends further support to previous studies relating deficient expression of *cry2* to depression in human patients (8) as well as in animal models of the disorder (23,44).

The present results imply that this deficient *cry2* expression potentially related to anxiety- and depression-like behavior in HAB mice can be rescued by chronic antidepressant pharmacotherapy. Since both, cry proteins and chronic fluoxetine administration, act to inhibit adenylyl cyclase (47,48) it can be speculated that modulation of cAMP production may form the common ground for the regulation at the molecular and behavioral level. Similarly, behavioral observations have led to propose that *cry2* may form part or regulate intracellular signaling pathways activated by chronic antidepressant drug treatment (46). A distinct effect of fluoxetine on the expression of *cry2* in “disease” (HAB) versus “control” (NAB) proposes *cry2* as a potential molecular element involved in mediating the differential behavioral consequences attributed to antidepressant treatment in patients and healthy subjects (49–51). Nevertheless, we have to admit that the observed alterations in hippocampal gene expressions may reflect genetic drift to phenotypes unrelated to anxiety-, depression- or sleeping behaviors. We tried to reduce effects of random genetic incidents by performing experiments in unrelated individuals of the HAB and NAB lines. Furthermore, we show in a pharmacological approach that chronic fluoxetine attenuates depression-like behavior (24) and normalizes aspects of deranged circadian locomotor activity in parallel with the hippocampal clock gene *cry2*, suggesting an association between these molecular markers and the behavioral phenotype beyond genetic drift. Yet, short-term selection and F2 panel association can indeed provide a more objective appraisal of anxiety-related compared to other risks.

The fact that augmented levels of hippocampal *per3* expression were also observed at the protein level and even detected in prefrontal cortical tissue indicates that differential expression of this clock gene in fluoxetine-treated HAB versus NAB mice is a particular robust phenomenon and potentially of central relevance to the observed circadian phenotype.

To conclude, we here firstly provide evidence that SSRI-based pharmacological antidepressant therapy acts to restore particular features of disrupted circadian rhythmicity in a genetic animal model presenting with high anxiety- and depression-related behavioral traits. The concomitant modulation of hippocampal clock gene expression further suggests an involvement of *per2*, *per3* and *cry2* as potential mediators of a shared molecular mechanism linking the therapeutic antidepressant response to fluoxetine-dependent modulation of circadian rhythms.

## Disclosure statement

The authors declare no conflict of interest. We would like to thank Irene Griesauer, Stefan Schulz, Msc, Deeba Khan, PhD and Stefanie Berger, MSc for their support throughout the course of experiments and data analysis.

## Funding information

D.D.P. is funded by the Austrian Science Fund (FWF): P22424 and P27520. N.S. is supported by the Austrian Science Fund (FWF): P22931-B18 and member of the special research network (SFB) 44.

## Supplementary Material

Supplementary_Figure_S2_corr.jpg
